# TRAIL stabilization and cancer cell sensitization to its pro-apoptotic activity achieved through genetic fusion with arginine deiminase

**DOI:** 10.18632/oncotarget.26398

**Published:** 2018-12-11

**Authors:** Elena Brin, Katherine Wu, Eleanor Dagostino, Mario Meng-Chiang Kuo, Yudou He, Wei-Jong Shia, Li-Chang Chen, Mariusz Stempniak, Richard Hickey, Robert Almassy, Richard Showalter, James Thomson

**Affiliations:** ^1^ Polaris Pharmaceuticals, San Diego, CA, USA

**Keywords:** TRAIL, arginine deiminase, apoptosis, fusion protein, anti-cancer biologic

## Abstract

Tumor necrosis factor (TNF)-related apoptosis-inducing ligand (TRAIL) binds to death receptors and induces apoptosis in various cancer cell lines while sparing normal cells. Recombinant TRAIL has shown good safety and efficacy profiles in preclinical cancer models. However, clinical success has been limited due to poor PK and development of resistance to death receptor-induced apoptosis. We have addressed these issues by creating a fusion protein of TRAIL and arginine deiminase (ADI). The fusion protein benefits from structural and functional synergies between its two components and has an extended half-life *in vivo*. ADI downregulates survivin, upregulates DR5 receptor and sensitizes cancer cells to TRAIL induced apoptosis. ADI-TRAIL fusion protein was efficacious in a number of cell lines and synergized with some standard of care drugs. In an HCT116 xenograft model ADI-TRAIL localized to the tumor and induced dose-dependent tumor regression, the fusion protein was superior to rhTRAIL administered at the same molar amounts.

## INTRODUCTION

TRAIL (also known as Apo2 ligand), is a trimeric protein, a TNF superfamily member, expressed as a type-II transmembrane protein and plays a physiological role in anti-tumor immune surveillance [1–6]. TRAIL induces apoptosis after binding to death receptor 4 (DR4 or TRAIL-R1) and/or death receptor 5 (DR5 or TRAIL-R2) [1, 2, 4–7]. Biologically active soluble TRAIL can be generated after cleavage at the stalk domain. TRAIL receptor agonists, soluble recombinant TRAIL and antibodies against DR4 and DR5 receptors, have been pursued as a promising anti-cancer strategy and showed favorable activity in pre-clinical studies [5–11]. In early clinical trials these agents showed good safety profile but had limited efficacy [7, 12].

One of the challenges hindering development of recombinant human TRAIL (rhTRAIL) is its very short half-life (3–5 minutes in mice and 30–60 minutes in primates; [13]). A number of groups addressed this issue through protein engineering (e.g. PEGylation, single chain Fc fusion, etc.) with the resulting molecules having improved PK with differential effect on activity ranging from less potent to more potent than the unmodified rhTRAIL trimer [14–20].

Many cancer cells are intrinsically resistant to TRAIL-induced apoptosis. Resistance can arise due to surface levels of TRAIL receptors (low levels of functional receptors and/or high levels of decoy receptors), modulation of pro- and anti-apoptotic signaling molecules such as cellular FLICE inhibitory protein (c-FLIP), inhibitors of apoptosis proteins (IAPs) and caspase 8 [1, 4–7, 21–25].

TRAIL can be sequestered by decoy receptors DcR1 (TRAIL-R3) and DcR2 (TRAIL-R4) and a soluble decoy receptor osteoprotegerin (OPG). DcR1 and DcR2 lack the functional death domain (DD), and therefore are unable to transmit the apoptotic signals induced by binding to TRAIL ligands [26–29].

Arginine deiminase (ADI) is an enzyme that converts arginine into citrulline and ammonia. Arginine deprivation can inhibit the growth of arginine auxotrophic cancers lacking key enzymes, such as argininosuccinate synthase (ASS1), that normal cells use to produce arginine from citrulline. A well tolerated arginine depleting drug, ADI-PEG 20, is currently being evaluated in clinical trials [30–32]. In an arginine auxotrophic cancer cell panel we observed synergy between ADI and TRAIL in inducing apoptosis in most of the tested cell lines including those that are otherwise resistant to rhTRAIL. We have explored potential mechanisms of synergy by examining the effect of ADI on the expression of TRAIL receptors and anti-apoptotic proteins such as survivin, IAPs, and c-Flip.

We have discovered that ADI derived from some species of Mycoplasma forms hexamers. Using *in silico* modeling we predicted structural complementarity between hexameric ADI and TRAIL with each modality stabilized when part of the fusion protein. We were able to produce multiple ADI-TRAIL fusion protein variants and have tested their activity *in vitro* and *in vivo*. This novel biologic has greatly improved half-life compared with rhTRAIL and shows promise as a cancer therapeutic in preclinical studies.

## RESULTS

### ADI sensitizes cancer cells to TRAIL-induced apoptosis

A number of cancer cell lines were treated with serially diluted ADI-PEG 20 or serially diluted rhTRAIL or the combination of the two (matrix design – each concentration of one agent tested with each concentration of the other agent). The treatment was carried for 72 h after which viable cell numbers were measured using resazurin and expressed as a percentage of the non-treated control as described in Materials and Methods. Percent reduction in relative cell viability achieved by each individual protein was compared to the effect of the combination of the two proteins (at the same concentrations). Highest Single Agent and Bliss Independence models [33] were used to analyse if there is any additivity, synergy, or antagonism between ADI-PEG 20 and rhTRAIL.

ADI-PEG 20 and rhTRAIL were synergistic or additive in most of the tested ADI-sensitive cancer cell lines as shown in Table 1 (some examples of synergy are shown in Supplementary Figure 1A–1D). Many of these cell lines were not sensitive to rhTRAIL and ADI-PEG 20 potentiated rhTRAIL activity.

**Table 1:** Effect of combining ADI-PEG 20 and TRAIL in ADI-sensitive cell lines

		Individual agent potency		ADI-PEG 20 and rhTRAIL Combination
Cancer	Cell line	ADI-PEG 20 EC50 (nM)	rhTRAIL EC50 (ng/mL)	
Prostate	PC3	1.3	>100	^**^
Ovarian	SKOV-3	0.7	>100	^**^
Pancreatic	Mia-Paca-2	1.0	30.0	^*^
	Panc-1	0.3	100.0	^***^
Colon	HCT116	0.9	5.8	^***^
	HT29	0.5	>100	^**^
Breast	MDA-MB-231	0.7	120.0	^**^
NSCLC	H1299	1.2	>100	^**^
Renal	786-O	1.4	>100	^**^
	ACHN	0.8	>100	^***^
	Caki-1	0.4	27.5	^***^
	Caki-2	0.7	>100	^***^
Melanoma	A375	0.9	>100	^**^
	SK-MEL-3	0.6	>100	^**^
	SK-MEL-24	1.0	>100	^–^
	MeWO	1.0	>100	^–^
	WM-115	2.0	>100	^***^
Glioblastoma	U87MG	0.8	>100	^**^
Burkitt’s Lymphoma	Ramos	1.5	>100	^*^
	Raji	0.4	80.0	^***^
	Daudi	0.2	>100	^–^
	NAMALWA	0.6	16.8	^*^
Leukemia	K562	0.3	>100	^***^
	MOLT4	0.9	>100	^*^
	HL60	10	>100	^**^
	Jurkat	0.9	1.9	^*^

^***^Synergy relative to each agent alone (CI = 0.4–0.74 by Bliss Independence Model).

^**^Slight synergy relative to each agent alone (CI = 0.75–0.9 by Bliss Independence Model).

^*^Additive relative to each agent alone (CI = 0.9–1 by Bliss Independence Model and < 0.9 by Highest Single Agent Model).

^–^Combination had same activity as the highest agent alone.

In addition to the above we have also analyzed the effect of ADI-PEG 20, rhTRAIL or their combination in select cell lines by flow cytometry analyzing cell viability and caspase 3/7 activation as described in Materials and Methods. Relative viability analysis, which can be affected by changes in proliferation and/or cell death, confirmed synergy or additivity between the two proteins. In the presence of ADI-PEG 20, rhTRAIL-induced apoptosis was increased in the tested cells (examples are shown in Supplementary Figure 1E–1F).

As expected in ASS1-high cancer cell lines that are not sensitive to ADI-PEG 20 the combination treatment had the same effect as rhTRAIL alone (data not shown).

### ADI upregulates TRAIL receptor DR5

One of the potential mechanisms by which ADI-PEG 20 sensitizes cancer cells to rhTRAIL could be through TRAIL surface receptor modulation. Therefore, we have analyzed TRAIL surface receptor levels after ADI-PEG 20 treatment using flow cytometry.

**Figure 1:**
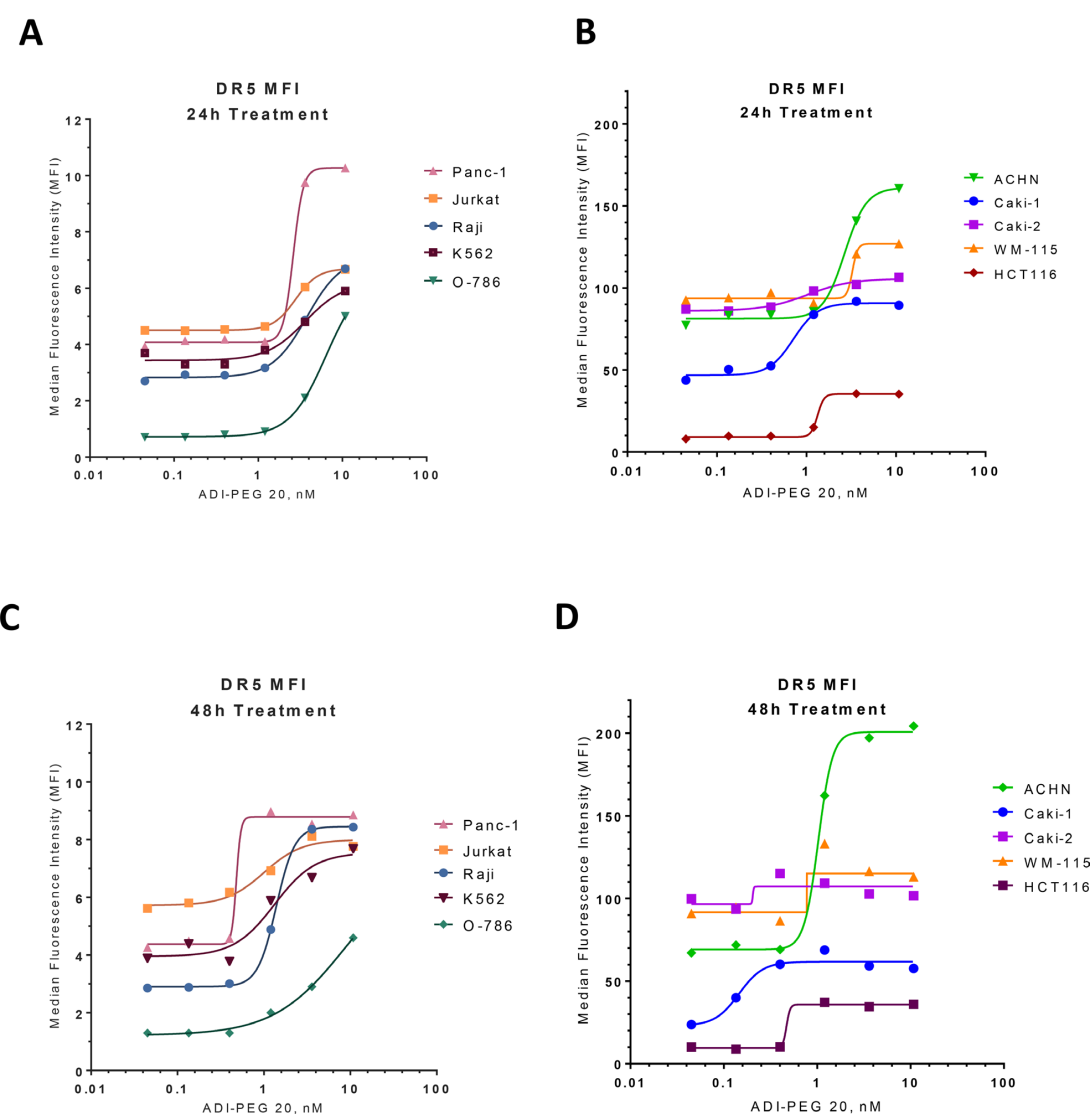
ADI up-regulates expression of TRAIL receptor DR5 Surface DR5 receptor levels were evaluated by FACS in various cancer cell lines following 24 h (**A**–**B**) and 48 h (**C**–**D**) treatment with ADI-PEG 20.

As shown on Figure 1, TRAIL receptor DR5 was upregulated on the surface of tested cell lines as soon as 24 h after treatment initiation (Figure 1A–1B) and remained upregulated after 48 h exposure to ADI-PEG 20 (Figure 1C–1D). Some of these cell lines (Panc-1, Jurkat, Raji, K562, O-786) had relatively low surface DR5 levels while others (ACHN, Caki-1, Caki-2, WM-115, HCT116) had relatively high surface DR5 which was further increased after ADI-PEG 20 treatment.

Analysis of other TRAIL receptors (DR4, DcR1 and DcR2) showed low or undetectable levels before and after ADI-PEG 20 treatment (data not shown).

We followed up with Western Blotting analysis to determine whether the increase in surface DR5 is due to an increase in total protein (rather than receptor translocation to the membrane). The total DR5 protein was indeed upregulated after ADI-PEG 20 treatment in all tested cell lines. DR4 was easily detectable by Western Blotting and ADI-PEG 20 had variable effect on its levels. After ADI-PEG 20 treatment (up to 48 h) DR4 total protein was not changed in Raji and HCT116 cells, it was increased in Jurkat, ACHN, Caki-1, Caki-2. In K562 DR4 decreased after ADI-PEG 20 treatment; this was observed in both Western Blotting and FACS experiments. In WM-115 DR4 was undetectable before and after treatment (data not shown).

### ADI-PEG 20 downregulates survivin

While TRAIL receptor levels are important for rhTRAIL activity they alone do not predict cancer cell line sensitivity to rhTRAIL. For example, as shown in Figure 1B and 1D, the ACHN cell line has relatively high surface DR5 but does not undergo apoptosis after rhTRAIL treatment (Table 1 and Supplementary Figure 1). Signaling molecules that inhibit caspase activation can block TRAIL-induced apoptosis. Thus, we investigated if ADI-PEG 20 treatment affects the caspase signaling pathway. Using Western Blotting analysis we evaluated levels of pro-survival proteins survivin, cIAP-1 and XIAP, as well as pro-caspase 8 and Smac in ten cell lines before and after ADI-PEG 20 treatment (24 h and 48 h).

**Figure 2:**
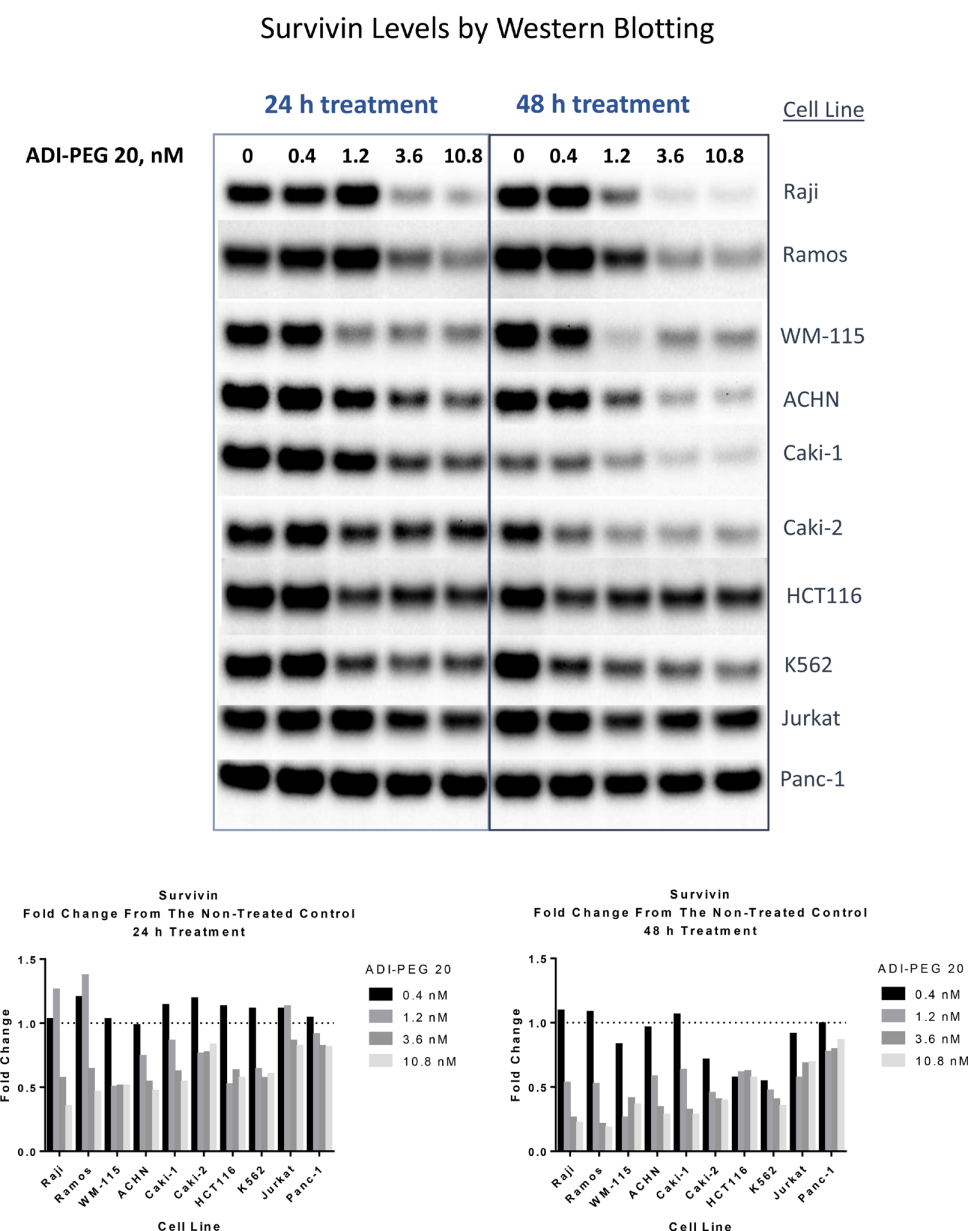
ADI treatment leads to reduction in survivin protein levels in ADI-sensitive cell lines Cell were collected and lysed following 24 h and 48 h treatment with ADI-PEG 20. Cell lysates were loaded with equal total protein content (20 µg), loading and Western transfer within the lysates from the same cell line were also verified with β-actin blots. Densitometry analysis relative to the non-treated control is shown in the graphs below the images of the Western Blotting data.

As shown in Figure 2 ADI-PEG 20 decreased survivin protein levels in all of the tested cell lines. In Panc-1 cells the decrease was very modest, but in the other tested cell lines the reduction in survivin protein was substantial, especially in Burkitt’s lymphoma cell lines Raji and Ramos, CML cell line K562, melanoma line WM-115, and renal cancer cell lines ACHN, Caki-1 and Caki-2.

Analysis of cIAP-1, XIAP and Smac revealed little to no change in these proteins across the tested cell lines (Supplementary Figures 2–3, data not shown). Changes in cFlip were not consistent across the cell lines, it decreased in K562 and Panc-1 while in other tested cell lines there was little to no change (data not shown).

Pro-caspase-8 was largely unaffected by ADI-PEG 20 treatment except for Burkitt’s lymphoma cell lines Ramos and Raji where ADI-PEG 20 induced activation of caspase 8 (Supplementary Figure 3).

### ADI-TRAIL fusion protein

Functional synergy between the ADI and TRAIL can be more effective *in vivo* if both localize to the tumor site. This can be achieved if the two proteins are linked together enabling the TRAIL moiety to retain the resultant biologic at the tumor site through receptor interaction.

Structural analysis of rhTRAIL and hexameric ADI variants lead us to hypothesize that a genetic fusion between ADI and TRAIL can result in a functional protein where both ADI and TRAIL are stabilized as a result of the fusion. In a fully assembled ADI-TRAIL fusion protein there are two TRAIL trimers per each ADI hexamer (Supplementary Figure 4).

We have expressed and purified a number of ADI-TRAIL fusion proteins, using several hexameric ADI variants (derived from different species) and various linkers (incorporated between ADI and TRAIL sequences; Supplementary Methods). Enzymatic activity of ADI was 10–20% improved when part of a fusion protein.

**Figure 3:**
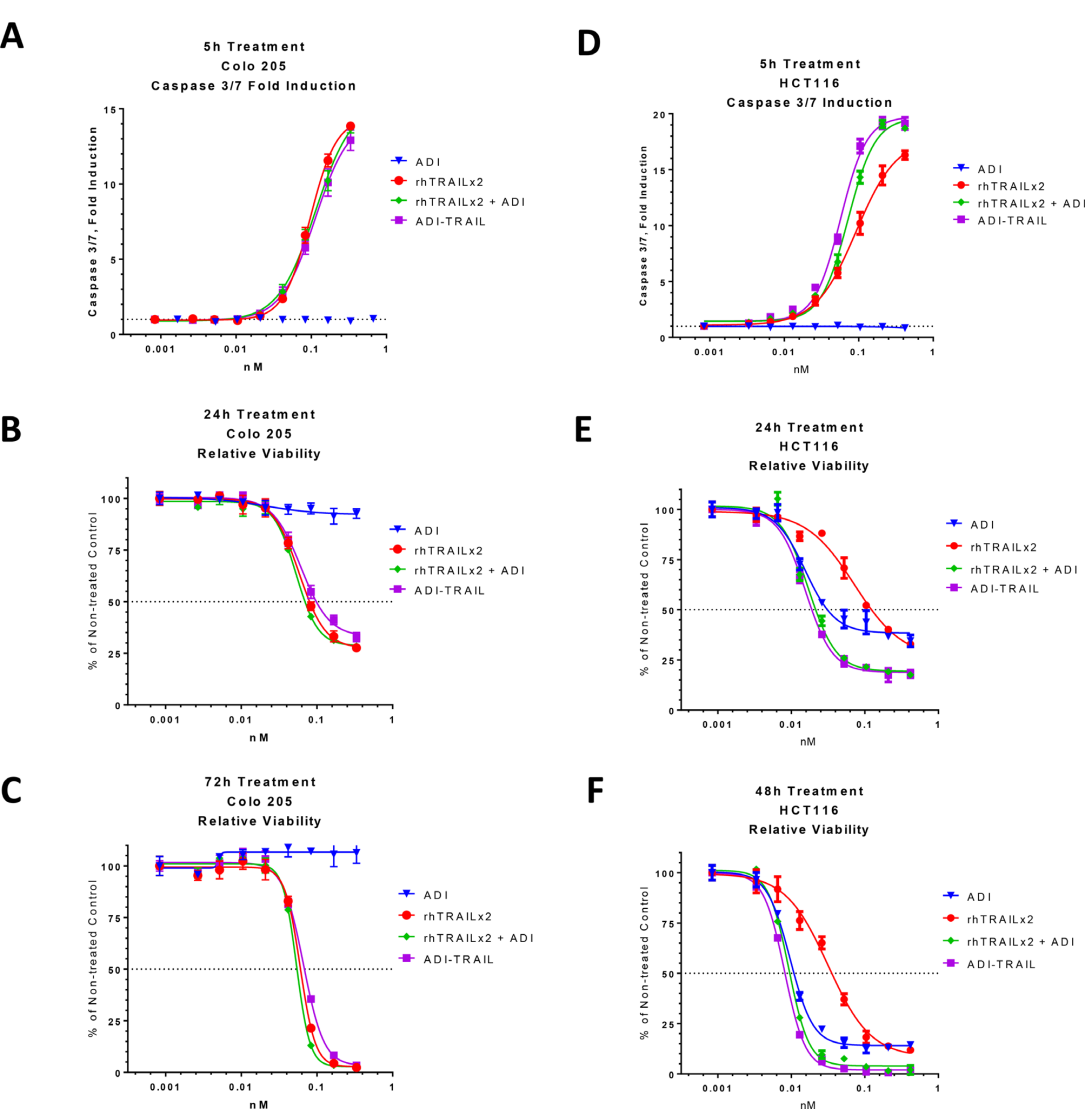
Activity of ADI-TRAIL fusion protein versus ADI and/or rhTRAIL Effect of ADI-TRAIL was compared to that of ADI + rhTRAIL versus ADI alone and rhTRAIL alone in ADI-non-sensitive cell line Colo 205 (**A**–**C**) and ADI-sensitive cell line HCT116 (**D**–**F**). Caspase 3/7 induction (A and D) was measured after 5 h treatment and relative cell viability was assessed after 24 h (B and E) and 48 h (F) and 72 h (C).

TRAIL activity was evaluated using Colo 205 cells. These cells express high levels of ASS1 and because of it are not affected by ADI treatment (Figure 3A–3C). Thus, we can use this cell line to measure the TRAIL activity in an ADI-TRAIL fusion protein without it being affected by the ADI moiety.

The effect of a representative ADI-TRAIL on caspase 3/7 activation after 5 h treatment is presented in Figure 3A and the effect on the relative viability of Colo 205 cells line after 24 h and 72 h treatment is presented in Figure 3B and 3C. The fusion protein had similar activity to rhTRAIL and to the combination of rhTRAIL and ADI (as separate proteins).

In addition, we evaluated ADI-TRAIL fusion proteins in an HCT116 cell line, which, as shown in Table 1 and Figure 3E–3F, is sensitive to both ADI and TRAIL (the two proteins are synergistic in this cell lines). By itself ADI does not induce caspase 3/7 activation in HCT116, however, it enhanced TRAIL-induced caspase 3/7 activation when combined with TRAIL as two separate proteins or in a fusion protein (Figure 3D). The combination of ADI and TRAIL as two separate proteins or as a fusion protein was more efficacious in reducing proliferation and viability of HCT116 cells than either protein alone. After 72 h of combined treatment viable cells were non-detectable while treatment with each individual protein was only partially effective.

We varied the ADI sequence (source species) or linker to produce a number of ADI-TRAIL fusion proteins and they generally had activities similar to the one used in Figure 3. In these fusion proteins TRAIL is linked to the C-terminus of ADI. When we switched the order and put TRAIL at the N-terminus and ADI at the C-terminus of the fusion protein TRAIL potency was somewhat improved (approximately 2-fold) as measured in Colo 205 cells (Supplementary Figure 5A–5C). However, the two fusion proteins, TRAIL-ADI and ADI-TRAIL, had similar potency and efficacy in inducing apoptosis of HCT116 cells (Supplementary Figure 5D–5F). From this and experiments combining ADI with various preparations of rhTRAIL (data not shown) it appears that ADI can enhance the TRAIL effect to a certain level and the combined effect of ADI and TRAIL is not significantly affected by small changes in the potency of the TRAIL moiety. In other words, we have observed a stronger synergy of ADI with a less potent preparation of TRAIL and the effect of the combination has some threshold which it reaches with either optimal or suboptimal preparations of TRAIL.

### ADI-TRAIL synergizes with standard of care drugs in pancreatic, renal and colon cell lines

We have evaluated the effect of combining ADI-TRAIL treatment with standard of care (SOC) drugs in cancer cell lines derived from pancreatic, colon and renal cancers. A summary of the data, which includes sensitivity (EC50 values in a relative viability assay) to ADI-TRAIL and presence or absence of synergy with SOC drugs, is shown in Table 2.

**Table 2:** Effect of combining ADI-TRAIL and standard of care drugs

Pancreatic Cancer Cell Line	ADI-TRAIL EC50, pM	irinotecan	5-FU	gemcitabine	cisplatin	mitomycin C	paclitaxel	docetaxel	erlotinib	sunitinib
Hs 700T	14.9	^*^	^*^	^-^	^**^	^*^	^**^	^*^	^*^	-
PANC-1	17.2	^**^	^***^	^**^	^**^	^*^	^**^	^***^	^*^	^**^
MIA PaCa-2	14.9	^**^	^**^	^**^	^**^	^**^	^***^	^***^	^***^	^***^
BxPC-3	170	^***^	^***^	^***^	^***^	^***^	^***^	^***^	^***^	^***^
L3.3	75.3	^**^	^**^	^*^	^**^	^***^	^**^	^**^	^*^	^***^

Colon Cancer Cell Line	ADI-TRAIL EC50, pM	irinotecan	5^-^FU	oxaliplatin	cobimetinib	regorafenib
HT29	19.6	^****^	^-^	^-^	^-^	^-^
HCT116	58.7	^**^	^**^	^**^	^**^	^**^
Colo205	113	^**^	^**^	^**^	^**^	^**^
DLD-1	31.6	^***^	^***^	^***^	^***^	^***^
Lovo	210	^***^	^**^	^***^	^**^	^***^

Renal Cancer Cell Line	ADI-TRAIL EC50, pM	cabozantinib	sunitinib
786-O	30.5	^**^	^*^
ACHN	31.5	^**^	^**^
Caki-1	34.4	^**^	^**^
Caki-2	31.3	^**^	^**^

^****^Strong synergy relative to each agent alone (CI < 0.4 by Bliss Independence Model).

^***^Synergy relative to each agent alone (CI = 0.4–0.74 by Bliss Independence Model).

^**^Slight synergy relative to each agent alone (CI = 0.75–0.9 by Bliss Independence Model).

^*^Additive relative to each agent alone (CI = 0.9–1 by Bliss Independence Model and < 0.9 by Highest Single Agent Model).

^-^Combination had same activity as the highest agent alone.

We observed additivity or synergy with at least one tested SOC drug in all the cell lines.

Among the tested pancreatic cell lines BxPC3 was the least sensitive (it also does not respond to ADI treatment), and in this cell line ADI-TRAIL synergized with all tested drugs – irinotecan, 5-FU, gemcitabine, cisplatin, mitomycin C, paclitaxel, docetaxel, erlotinib and sunitinib. Across all five tested pancreatic cell lines paclitaxel and docetaxel were the most synergistic with ADI-TRAIL suggesting that ADI-TRAIL combination with tubulin inhibitors could be effective in pancreatic cancer treatment.

Irinotecan was synergistic with ADI-TRAIL in all five tested colon cancer cell lines, while 5-FU and oxaliplatin were synergistic in 4 out of 5 cell lines.

The combination of ADI-TRAIL and cabozantinib was synergistic in all four renal cell lines tested, while the combination with sunitinib was synergistic in three cell lines and additive in the fourth.

### ADI-TRAIL activity in the HCT116 xenograft model

Next, we compared the anti-tumor activity of ADI-TRAIL fusion protein with that of the individual proteins (ADI and TRAIL) in the HCT116 xenograft mouse model. Female athymic Nude mice were inoculated with HCT116 cells subcutaneously. On Day 7 post inoculation mice were randomized into four treatment groups (5 mice per group) and administered rhTRAIL, ADI, ADI-TRAIL fusion protein or vehicle control (PBS buffer) by intravenous injection. Treatment with rhTRAIL was performed daily for 5 consecutive days (Days 7–11 post tumor implantation) with a 5 mg/kg dose. ADI was dosed at 20 mg/kg and ADI-TRAIL fusion protein at 30 mg/kg (same molar amount of ADI in the two groups; 30 mg of ADI-TRAIL contains 20 mg of ADI moiety and 10 mg of TRAIL moiety), both on Days 7 and 15 post tumor implantation.

**Figure 4:**
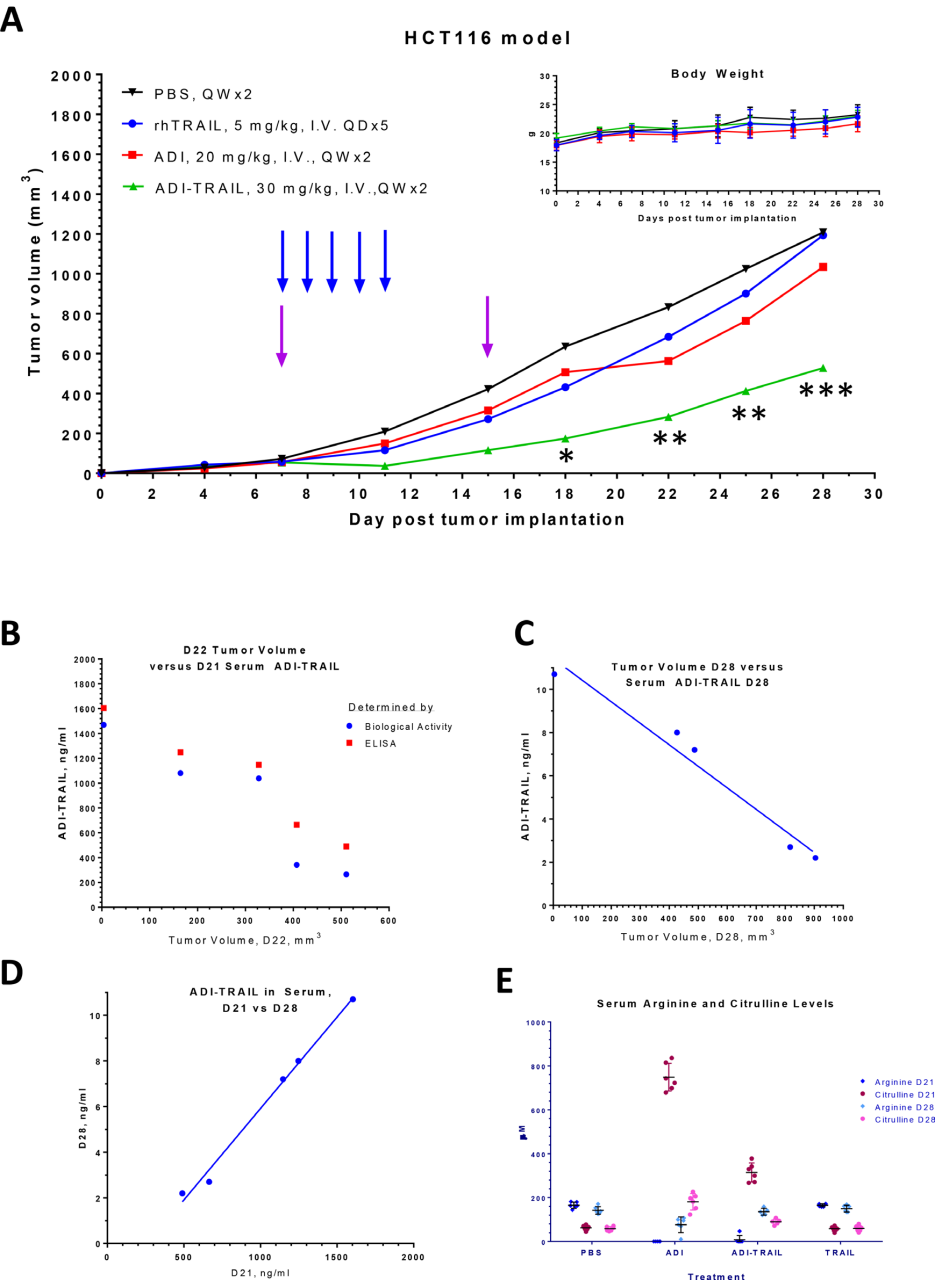
ADI-TRAIL is efficacious in HCT116 xenograft model Mice were treated with rhTRAIL, ADI or ADI-TRAIL. In all groups mice maintained their body weight (A inset), reduced tumor growth was observed in ADI-TRAIL treatment group (**A**). Arrows indicate when the treatment was administered, blue for rhTRAIL and purple for the other three groups. The statistical significance of the tumor reduction in the fusion protein treated group as compared to the vehicle treated control group was assessed by the 2-way ANOVA, ^*^p < 0.05, ^**^p < 0.01, ^***^p < 0.001. Serum was taken on Days 21 and 28 post tumor implantation (Day 6 and 13 post last treatment). ADI-TRAIL concentration was measured using biological assay (B) and ELISA (**B**–**D**), and was found to be inversely correlated with the tumor volume (B–C). Arginine and citrulline levels were measured by LC/MS/MS (**E**).

ADI, rhTRAIL, or the fusion protein did not cause any noticeable weight loss. ADI slowed tumor growth only slightly (not statistically significant), rhTRAIL initially inhibited tumor growth but after the dosing was stopped tumors resumed growth as rhTRAIL would have been cleared out due to its short half-life (3–5 minutes in mice [13]). After the first dose of ADI-TRAIL we noted tumor regression in all treated animals, though by the time of the second dose the tumors started to regrow; the second dose slowed their growth. Comparison with the vehicle treated control group by the 2-way ANOVA showed statically significant reduction in the tumor growth in the fusion protein treated group but not in the other two treatment groups (Figure 4A).

Serum was taken on Days 21 and 28 post tumor implantation, or Days 6 and 13 post last treatment. We measured ADI-TRAIL levels in serum using both ELISA and cell-based assays (see Materials and Methods). The results from the first time point for total fusion protein concentration measured by ELISA and biologically active protein measured by the biological activity assay were very similar to one another indicating that most of the serum ADI-TRAIL was biologically active 6 days after the last administration. ADI-TRAIL serum levels 13 days after the last injection were too low to measure by the biological assay but were detectable by ELISA. The concentration decreased about 200-fold in 7 days (Figure 4D), which indicates that the half-life was approximately 21 hours. As shown in Figure 4B–4C, serum ADI-TRAIL inversely correlated with the tumor volume. Arginine and citrulline levels in these serum samples are shown in Figure 4E. Arginine was completely depleted on Day 21 (6 days post last treatment) in ADI treatment group and in all but one animal in the ADI-TRAIL group (this one mouse with low but detectable arginine had the largest tumor volume). On Day 28 serum arginine in the ADI-TRAIL group was similar to the vehicle control and rhTRAIL groups; arginine was measurable in the ADI group but was still reduced compared with the control group. Serum citrulline levels were higher in the ADI treatment group compared with the ADI-TRAIL group at both time points (on Day 28 citrulline in ADI-TRAIL group was only slightly elevated compared with the control group). Lower arginine and higher citrulline imply higher concentration of ADI versus ADI-TRAIL in the serum. The stability of the two proteins should be similar. Thus, we hypothesized that the apparent difference in serum concentration between ADI and ADI-TRAIL is due to the fusion protein localization to the tumor site (through the TRAIL moiety) thereby decreasing its serum levels. We tested this hypothesis in the follow-up HCT116 xenograft study described below.

**Figure 5:**
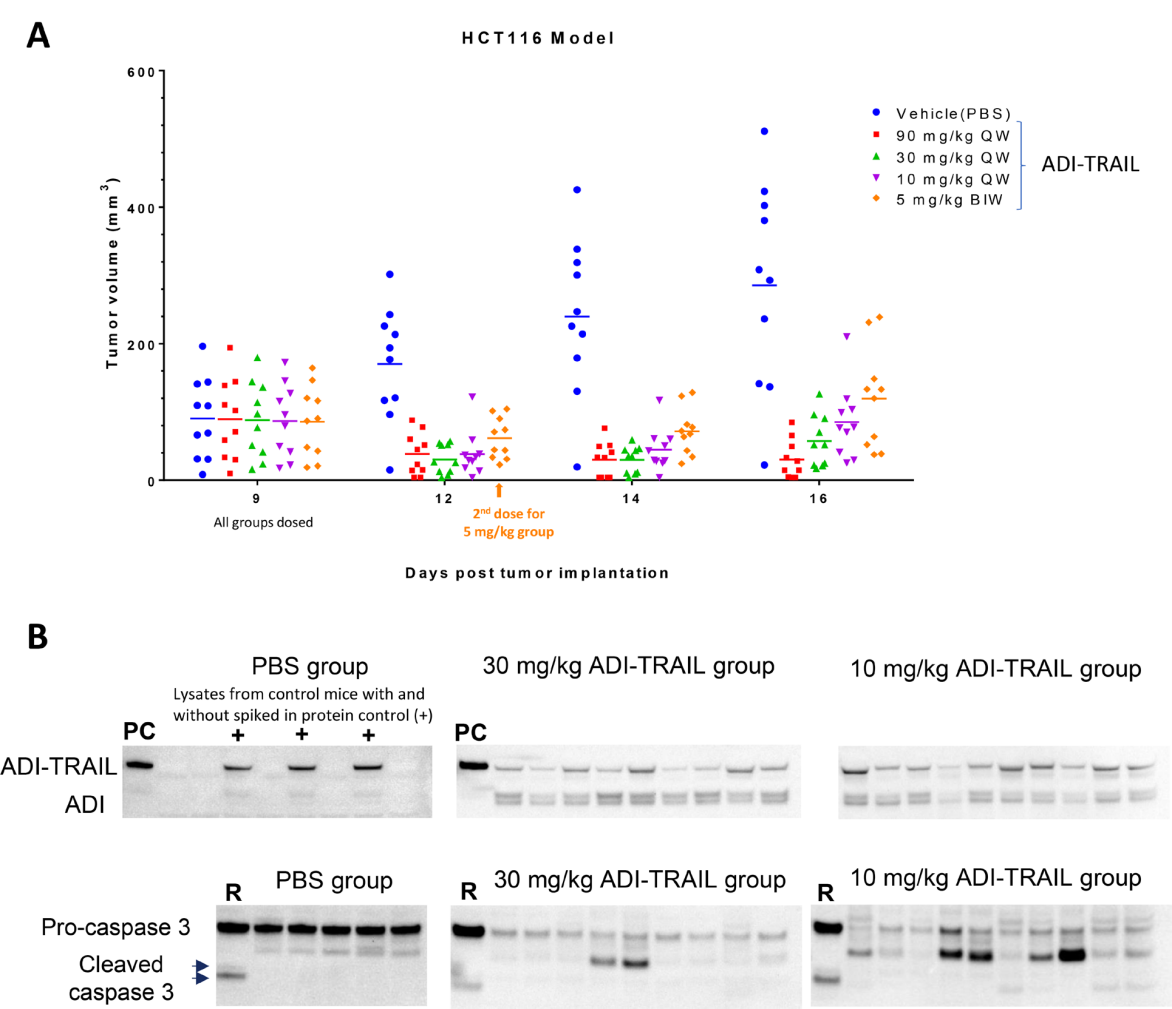
ADI-TRAIL induces dose-dependent tumor growth reduction in HCT116 xenograft model and localizes to the tumor site Mice were randomized into treatment groups on Day 9 post tumor implantation and injected I.V. with vehicle control or ADI-TRAIL at 5, 10, 30 or 90 mg/kg. The 5 mg/kg group was treated again on Day 12 (3 days post initial treatment). Tumor volumes were measured on Days 9, 12, 14 and 16 post tumor implantation (**A**) and 2-way ANOVA was used to evaluate statistical significance of tumor volume difference between the treatment groups and control group. *P* values for all three treatment groups versus vehicle control were equal (5 mg/kg group on Day 12) or less than 0.0001. On Day 16 (Day 7 post treatment initiation) there were also statistically significant differences between high and low dose groups: 90 mg/kg group versus 10 mg/kg group *p* = 0.0477, 90 mg/kg group versus 5 mg/kg group *p* = 0.0014, 30 mg/kg group versus 5 mg/kg group *p* = 0.0247. Mice were injected with ADI-TRAIL one more time and their tumors were collected and snap frozen. The tumors were then homogenized, lysed and analyzed by Western Blotting (**B**) for the presence of ADI-TRAIL using anti-ADI antibody and caspase 3 using antibody that recognizes pro- and active forms of caspase 3. Tumor lysates from individual mice were normalized by protein content (16 µg). ‘PC’ – protein control (5 ng recombinant ADI-TRAIL). ‘+’ - 5 ng recombinant ADI-TRAIL was spiked into tumor lysate from a vehicle control mouse. ‘R’ –Lysate from Raji cells treated with ADI-TRAIL.

Mice were randomized into five treatment groups (10 mice per group) on Day 9 post HCT116 inoculation (similar starting tumor volumes between the groups) and administered different doses of ADI-TRAIL fusion protein or vehicle control (PBS buffer) by intravenous injection. ADI-TRAIL dose groups were as follows: 90 mg/kg, 30 mg/kg, 10 mg/kg, and 5 mg/kg. The first three groups were dosed only on Day 9 and the 5 mg/kg group was dosed on Day 9 and Day 12 post tumor implantation. Tumors regressed after the dosing in all but two ADI-TRAIL treated mice in the low dose group as measured on Day 3 post dosing. The tumor volume stayed low at the next measurement on Day 5 post treatment initiation. A week after the dosing, tumors in the 90 mg/kg group were still not growing while in the other groups they had started to regrow (Figure 5A). Dosing once with 10 mg/kg appeared to be more effective than twice with 5 mg/kg (not statistically significant). Analysis by 2-way ANOVA showed that tumor volume reduction after ADI-TRAIL treatment was statistically significant. *P* values for treatment group versus vehicle control on Day 12 were *p* < 0.0001 for the 10 mg/kg, 30 mg/kg, and 90 mg/kg groups and *p* = 0.0001 for the 5 mg/kg group; on Days 14 and 16 *p* < 0.0001 for all groups. On Day 16 (Day 7 post treatment initiation) there were also statistically significant differences between high and low dose groups: 90 mg/kg group versus 10 mg/kg group *p* = 0.0477, 90 mg/kg group versus 5 mg/kg group *p* = 0.0014, 30 mg/kg group versus 5 mg/kg group *p* = 0.0247.

Mice in the 10 and 30 mg/kg groups were dosed one more time and terminated the next day, their tumors were collected and snap frozen. The tumors were lysed as described in Materials and Methods and analyzed by Western Blotting with anti-ADI and anti-caspase 3 antibodies. Tumor lysate somewhat obscures detection of the fusion protein – when we spiked ADI-TRAIL into the lysate of tumors from vehicle treated mice the intensity of ADI-TRAIL band decreased approximately two-fold compared with the same amount of the protein in the absence of a tumor lysate. Nevertheless, as shown in Figure 5B ADI-TRAIL was detected in all tumor lysates from the treated mice. Pro-caspase 3 levels were similar in tumor lysates from vehicle treated animals and decreased in all ADI-TRAIL treated mice. These data indicate that ADI-TRAIL localizes to mouse tumors and induces apoptosis within 24 h after administration.

## DISCUSSION

TRAIL receptor agonists including soluble rhTRAIL (Dulanermin, Amgen) have been explored as anti-cancer therapeutics due to their ability to induce cancer cell apoptosis while sparing normal cells. They showed a good safety profile but unfortunately efficacy was lacking [35–41]. Dulanermin has a very short serum half-life of 20 minutes [19]. In addition to short half-life, development of resistance to TRAIL-induced apoptosis may have also contributed to poor efficacy.

To overcome the hurdles facing Dulanermin we have produced a fusion protein between hexameric ADI and TRAIL. This fusion protein has a greatly improved PK profile (17–21 h half-life in mice versus 3–5 min for rhTRAIL), benefits from functional synergy between ADI and TRAIL, and has potent anti-cancer activities *in vitro* and *in vivo* (Figures 3–5).

As shown in Table 1 and Supplementary Figure 1, ADI sensitizes cancer cells to TRAIL enabling them to overcome resistance to TRAIL induced apoptosis. Another group [34] showed that the combination of ADI-PEG 20 and rhTRAIL inhibited growth of four melanoma cell lines *in vitro* better than either single agent alone. They observed upregulation of DR4 and DR5 receptors and downregulation of survivin in two melanoma cell lines. In a panel of ten ADI-sensitive cancer cell lines following ADI treatment we have observed upregulation of TRAIL receptor DR5 (Figure 1) in all cell lines. Depending on the cell line, DR4 receptor was either not changed, upregulated or downregulated (in one cell line K562) (data not shown) and survivin was decreased, with the extent of decrease varying across the cell lines (Figure 2). Inhibitors of apoptosis XIAP, IAP-1 and IAP-2 were decreased in only some of the tested cell lines (Supplementary Figures 2–3).

In addition to DR5 upregulation and downmodulation of survivin, ADI induces cell cycle arrest and ROS generation ([42–43], data not shown), which may further sensitize cells to TRAIL [44–45].

Other anti-cancer drugs can upregulate DR5 and sensitize cancer cells to TRAIL [46–50].

To promote TRAIL induced cancer cell apoptosis and reduce the risk of resistance, SOC drugs can be combined with the ADI-TRAIL fusion protein. Here, we investigated a few SOC drugs typically used to treat patients with pancreatic, colon and renal cancers. We found that many of these drugs are synergistic with ADI-TRAIL in most of the tested cell lines (Table 2). In particular, combination of ADI-TRAIL was synergistic with the tubulin inhibitors paclitaxel and docetaxel in the five tested pancreatic cancer cell lines, with irinotecan in all five tested colon cancer cell lines, and with cabozantinib in all four tested renal cell lines.

The fusion protein can be localized to tumors expressing TRAIL receptors. Indeed, we have detected ADI-TRAIL in tumor lysates from the ADI-TRAIL mice (Figure 5) similar to other TRAIL receptor agonists [18]. Tumor localization of the fusion protein should enhance ADI moiety anti-cancer activity *in vivo* via local arginine depletion (rather than systemic depletion with the enzyme by itself).

Identification of biomarkers of sensitivity to ADI-TRAIL beyond ASS1 and TRAIL receptors will help identify patients that are most likely to benefit from ADI-TRAIL treatment. While upregulation of TRAIL receptor levels often leads to increased sensitivity of a cancer cell line to TRAIL-induced apoptosis comparing receptor levels on different cancer cells has only limited predictive value as can be seen from the data presented in Table 1 and Figure 1A–1D, and published data from other groups [24]. In other words, surface expression of DR5 and/or DR4 receptors are required but not sufficient for cancer cell sensitivity to TRAIL induced apoptosis.

Future studies will further characterize the therapeutic potential of ADI-TRAIL such as its ability to penetrate tumors with dense stroma (e.g. pancreatic ductal adenocarcinoma). And with ADI being a foreign protein we need to investigate whether expected antibody response to ADI moiety will have positive and/or negative consequences. Anti-ADI antibodies can expedite the clearance of the protein; while antibody binding to the ADI moiety in the context of ADI-TRAIL bound at the tumor site can result in ADCC mediated tumor killing. It is worth noting that the clinically tested *M. homini*s ADI-PEG 20 is effective (and safe) in patients.

In summary, the novel anti-cancer biologic ADI-TRAIL fusion protein benefits from structural and functional complementarities/synergies of its components and has promising anti-cancer activity *in vitro* and *in vivo*. An expanded range of cancers are likely to respond to the fusion protein versus each protein alone, and in tumors sensitive to each individual protein the fusion protein should have better efficacy (than its individual protein components). Potency and efficacy of the fusion protein are likely to be further enhanced when combined with other anti-cancer therapies.

## MATERIALS AND METHODS

### Reagents

ADI-PEG 20 is a recombinant protein cloned from *Mycoplasma* and subsequently produced in *Escherichia coli*. This recombinant dimeric protein is PEGylated with 20,000 MW PEG [51]. ADI-TRAIL is a fusion protein consisting of recombinant human TRAIL and recombinant hexameric ADI. The fusion pr otein was prepared as described in the Supplementary Methods. All data shown for native ADI was obtained with hexameric ADI. Cancer cell lines were purchased from ATCC. IFNγ, Live/Dead fixable stains (Green and Near-IR), CellEvent Caspase 3/7 kit, Anti-DcR2-FITC, SimplyBlue^™^ SafeStain, BCA assay kit, Blocker Casein, mouse anti-GAPDH monoclonal antibody HRP conjugate, SuperSignal West Femto maximum sensitivity substrate, RIPA buffer, and protease inhibitors were obtained from ThermoFisher Scientific. The following flow cytometry reagents were acquired from BD Bioscience: SB(BSA) buffer, anti-DcR1 mouse PE anti-human DcR1, APC Mouse IgG1 κ Isotype control (clone MOPC-21), PE Mouse IgG1 κ Isotype Ctrl (clone MOPC-21), FITC mouse IgG1 κ Isotype Ctrl (clone MOPC-21). Anti-DR4-APC (clone DJR1) and anti-DR5-PE (clone DJR2-4) were purchased from BioLegend.

TMB substrate and stop solution were purchased from Sigma.

Most antibodies used for Western Blotting were purchased from Cell Signaling Technology; cFlip specific antibody was from Adipogen. Resazurin and rhTRAIL were from R&D Systems.

Goat anti-mouse IgG-HRP was from Santa Cruz Biotech.

CellTiter-Fluor, CellTiter-Glo, and Caspase 3/7 Glo were obtained from Promega.

### Cancer cell viability and caspase assays

Cancer cell lines were cultured according to manufacturer’s instructions. Cells were treated with increasing concentrations of ADI-PEG 20 and analyzed after 24 h, 48 h or 72 h incubation.

Cell viability was assessed using resazurin, CellTiter-Fluor, or CellTiter-Glo. Relative cell viability was calculated by dividing the cell viability signal from a test sample by that of a non-treated control. For evaluation of apoptosis induction, Caspase 3/7 activation was assessed using Promega’s caspase 3/7 Glo reagent with luminescence read by a plate reader. Caspase 3/7 activation and cell viability were also assessed by flow cytometry analysis of cells stained with fluorescent reagents detecting activated caspase 3/7 and dead cells (CellEvent Caspase 3/7 kit).

Live/Dead stain and antibodies recognizing TRAIL receptors were incubated for 30 minutes on ice. Unincorporated Live/Dead dye and unbound antibodies were washed away, and cells were analyzed by a multi-color flow cytometer. Live/dead stain and antibodies were labeled with distinct fluorophores detected in different channels of a flow cytometer. Isotype control antibodies were used to assess and control for non-specific binding. Receptor expression was analyzed in a cell population gated on singlet and live cells.

### Analysis of TRAIL receptor surface levels in cancer cell lines

Cell were collected and washed in cold SB(BSA) buffer. The cells were then resuspended in SB(BSA) buffer at 2 × 10^6^ cells/mL and incubated at room temperature for at least 10 min. Each sample was split in four (50 µL each) for specific staining with anti-DR-4 and anti-DR-5 mAbs, anti-DcR1 and anti-DcR2 mAbs and to assess non-specific binding with isotype control antibodies. Live/Dead fixable green stain was prepared according to manufacturer’s instructions and the solubilized stain was used at a final dilution of 1:500. Live/Dead stain and antibody mixtures were incubated with samples on ice protected from light for 30 min, washed twice in SB(BSA) buffer and resuspended in 150 µL of SB(BSA) buffer. Non-stained and single stained controls were used to adjust instrument settings. Ten thousand events were acquired for each sample on a Guava EasyCyte 8 or 12HT (Millipore). Data acquisition and analysis were performed with InCyte software (Millipore). Cells were gated on singlets (determined by FSC-H versus FSC-A) and live cells within the singlet gate were analyzed for DR-4, DR-5, DcR1 and DcR2 levels.

### Analysis of combined effect of two test agents

Highest Single Agent and Bliss models were used to analyze for additivity, synergy, or antagonism between the two test agents as described in Foucquier and Guedj’s review [33].

### Western blotting

Cells were lysed in RIPA buffer supplemented with protease inhibitors. Tumors were homogenized with a BeadBug mini homogenizer from Benchmark Scientific according to manufacturer’s instructions. Lysate protein concentrations were determined by BCA assay and ∼20 µg of protein was loaded for each sample onto 4–12% Bis-Tris polyacrylamide gels, two identical gels were used – one was stained with Simply Blue to confirm the protein amount loaded and another was transferred onto PVDF membrane with an iBlot 2 Western Blotting System (Thermo-Fisher Scientific). The membranes were blocked with 0.5% non-fat dry milk-TBST and probed with primary antibodies followed by incubation with secondary antibody conjugated with HRP using SNAP i.d. system (EMD Millipore). Housekeeping control protein β-actin was detected with antibody directly conjugated to HRP and used to verify even Western Blotting transfer and protein loading with samples derived from the same cell line. Detection was conducted with SuperSignal West Femto maximum sensitivity substrate and both the blots and stained protein gels were imaged on a BioRad’s ChemiDoc Imager and band intensities were quantified using Image Lab software.

### Mouse studies

Female athymic Nude mice were inoculated with HCT116 cells subcutaneously. On Day 7 post inoculation mice were randomized into treatment groups (to have similar starting tumor volumes between the groups) and administered rhTRAIL, ADI, ADI-TRAIL fusion protein or vehicle control (PBS buffer) by intravenous injection. Treatment with rhTRAIL was performed daily for 5 consecutive days (Days 7–11 post tumor implantation). ADI and ADI-TRAIL fusion proteins were injected on Days 7 and 15 post tumor implantation.

Arginine and citrulline levels in serum were measured by LC/MS/MS.

The mouse studies were performed at Explora BioLabs in accordance with their guidelines.

### ELISA for determination of ADI-TRAIL in mouse sera

High binding 96-well flat bottom plates (Costar) were coating overnight at 4°C with 2.5 µg/mL anti-TRAIL mAb. The next day plates were washes three times with PBS-0.1% Tween-20 (PBST) and blocked with Blocker Casein (ThermoFisher Scientific) for 2 hours at room temperature on a shaker followed by another three washes with PBST. Mouse serum samples and purified recombinant ADI-TRAIL standard were serially diluted in Blocker Casein and added to the plate. Sample incubation was carried out for 2 h on a shaker at room temperature. After incubation was completed plates were washed three times with PBS-0.1% Tween-20. Anti-ADI polyclonal rabbit antibody diluted in Blocker Casein with 0.1% Tween 20 was then added to the plates and incubated for 1 h at room temperature on a shaker. Plates were washed three times with PBST and incubated with anti-rabbit IgG-HRP diluted in Blocker Casein with 0.1% Tween 20 at room temperature for 1 h on a shaker. After the incubation plates were washed five times with PBST, then incubated with TMB substrate for 3 minutes after which the reaction was stopped with stop solution. Absorbance was determined in a plate reader at OD450 nm. The ADI-TRAIL concentration in mouse serum samples was determined based on a standard curve. The standard curve was fitted using log-log linear regression in GraphPad.

### Biological assay for detection of active ADI-TRAIL in mouse sera

Sera from treated mice and recombinant purified ADI-TRAIL were diluted in cell culture media and added to plates with HCT116 or Colo 205 cells plated the day before. CellTiter-Glo was used to determine caspase 3/7 activation after 5 h incubation and CellTiter-Fluor was used to determine relative viability after 48 h incubation. Serially diluted ADI-TRAIL was used as a standard to interpolate the concentration of the fusion protein in serum samples.

### Statistical analysis

Two-way ANOVA was performed to compare different treatments using GraphPad.

## SUPPLEMENTARY MATERIALS FIGURES


