# Supplementary material for: TRAIL stabilization and cancer cell sensitization to its pro-apoptotic activity achieved through genetic fusion with arginine deiminase
Source: Oncotarget. 2018 Dec 11;9(97):36914–28. doi: 10.18632/oncotarget.26398 (PMC6319333; doi:10.18632/oncotarget.26398)
Supplement: Supplementary file 1 [file oncotarget-09-36914-s001.pdf]

TRAIL stabilization and cancer cell sensitization to its pro-apoptotic activity achieved through genetic fusion with arginine deiminase

SUPPLEMENTARY MATERIALS

MATERIALS AND METHODS

ADI and ADI-TRAIL expression and purification

pET21a (Novagen) expression vector was used for both ADI and ADI-TRAIL genes. The fusion of ADI and hrTRAIL (114–281) was facilitated by the incorporation of a linker between the carboxy terminal residue of ADI and the amino terminal residue of TRAIL, for the representative fusion protein used in the experiments described in the manuscript a flexible GGGGS linker was used.

Proteins were expressed in E.coli BL21(DE3) (Invitrogen) in Terrific Broth auto induction media, in baffled Erlenmeyer flasks. Cells were grown at 30–37° C overnight at 250 rpm in a shaking incubator. Cell pellets were harvested by centrifugation at 10,000 rpm for 20 minutes and then stored at −80° C for purification conducted as described below.

Cell pellets were re-suspended in 20 mM NaPO4 pH 8.5, 20 mM imidazole, at a ratio of 5 mL of buffer per gram of cell paste. The cells were then lysed using an M110L microfluidizer (Microfluidics, Westwood MA). Insoluble material was removed by centrifugation at 10,000 rpm. The supernatant was loaded onto a 50 mL NiNTA Superflow (Qiagen) column equilibrated with 20 mM $NaPO_4$ pH 8.5, 20 mM imidazole, using an AKTA FPLC (GE Amersham Pharmacia). The column was washed to baseline with 20 mM $NaPO_4$ pH 8.5, 20 mM imidazole and then the bound protein was eluted with: for ADI - a step gradient of 20 mM $NaPO_4$ pH 8.5 500 mM imidazole; for ADI-TRAIL - a 0–100% linear gradient of 20 mM $NaPO_4$ pH 8.5 to 20 mM $NaPO_4$ pH 8.5, 50 mM imidazole over 10 column volumes. The eluted protein was then bound to a 50 mL Q-Sepharose Fast Flow (GE Amersham Pharmacia) column and washed to baseline with 20 mM $NaPO_4$ pH 8.5. The bound ADI was eluted with a linear gradient of 20 mM $NaPO_4$ pH 8.5 to 20 mM $NaPO_4$ pH 8.5, 1M NaCl over 10 column volumes. ADI-TRAIL was eluted with a 0–100% linear gradient of 20 mM $NaPO_4$ pH 8.5 to 20 mM $NaPO_4$ pH 8.5, 1 M NaCl over 10 column volumes. Peak fractions were pooled, made 1 M with ammonium sulfate, and then loaded onto a 50 mL Phenyl Sepharose High Performance (GE Amersham Pharmacia) column. The column was washed to baseline with 20 mM $NaPO_4$ pH 8.5, 1 M ammonium sulfate then the bound protein was eluted with a linear gradient of 20 mM $NaPO_4$ pH 8.5, 1 M ammonium sulfate to 20 mM $NaPO_4$ pH 8.5 over 10 column volumes. The peak fractions were pooled, concentrated to ~20 mL or less in an Amicon stir cell concentrator with 10 kDa MWCO Ultracel® membrane (EMD Millipore, Billerica MA), sterile filtered through a 25 mm 0.2 micron Supor® syringe filter (Pall Life Sciences), then loaded onto a 500 mL Superdex 200 size exclusion column (GE Amersham Pharmacia). The column was developed at 1 mL/min in 10 mM HEPES pH 7.5 150 mM NaCl buffer. Peak fractions were pooled and concentrated to ~1 mg/mL and stored at −80° C.

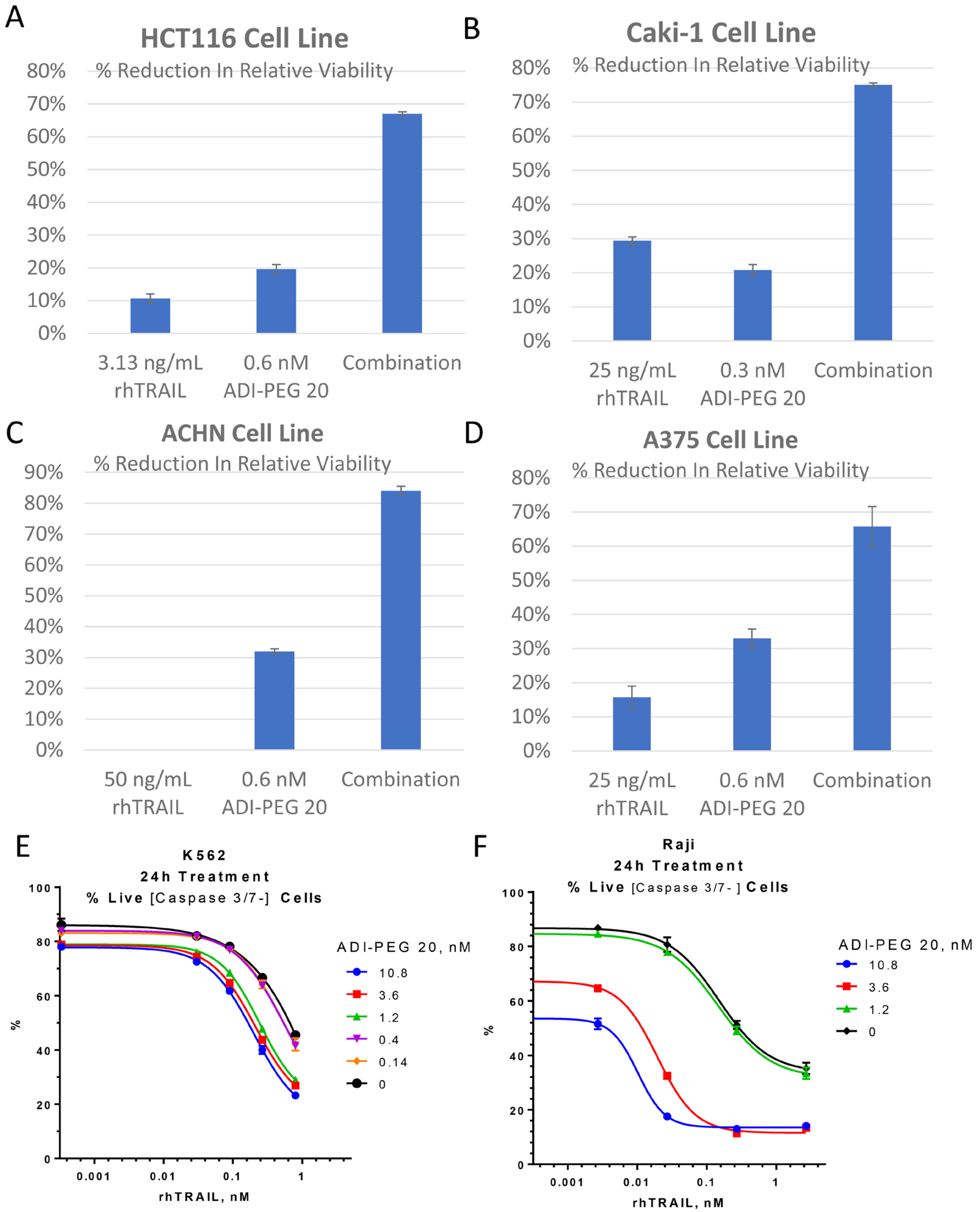

Supplementary Figure 1: Examples of synergy between ADI-PEG 20 and rhTRAIL. The combination of ADI-PEG 20 and rhTRAIL in HCT116 (**A**), Caki-1 (**B**), ACHN (**C**), A375 (**D**), K562 (**E**), Raji (**F**) had a much greater effect on reduction in relative cell viability (A–D) and % Live cells (E–F) than either individual protein.

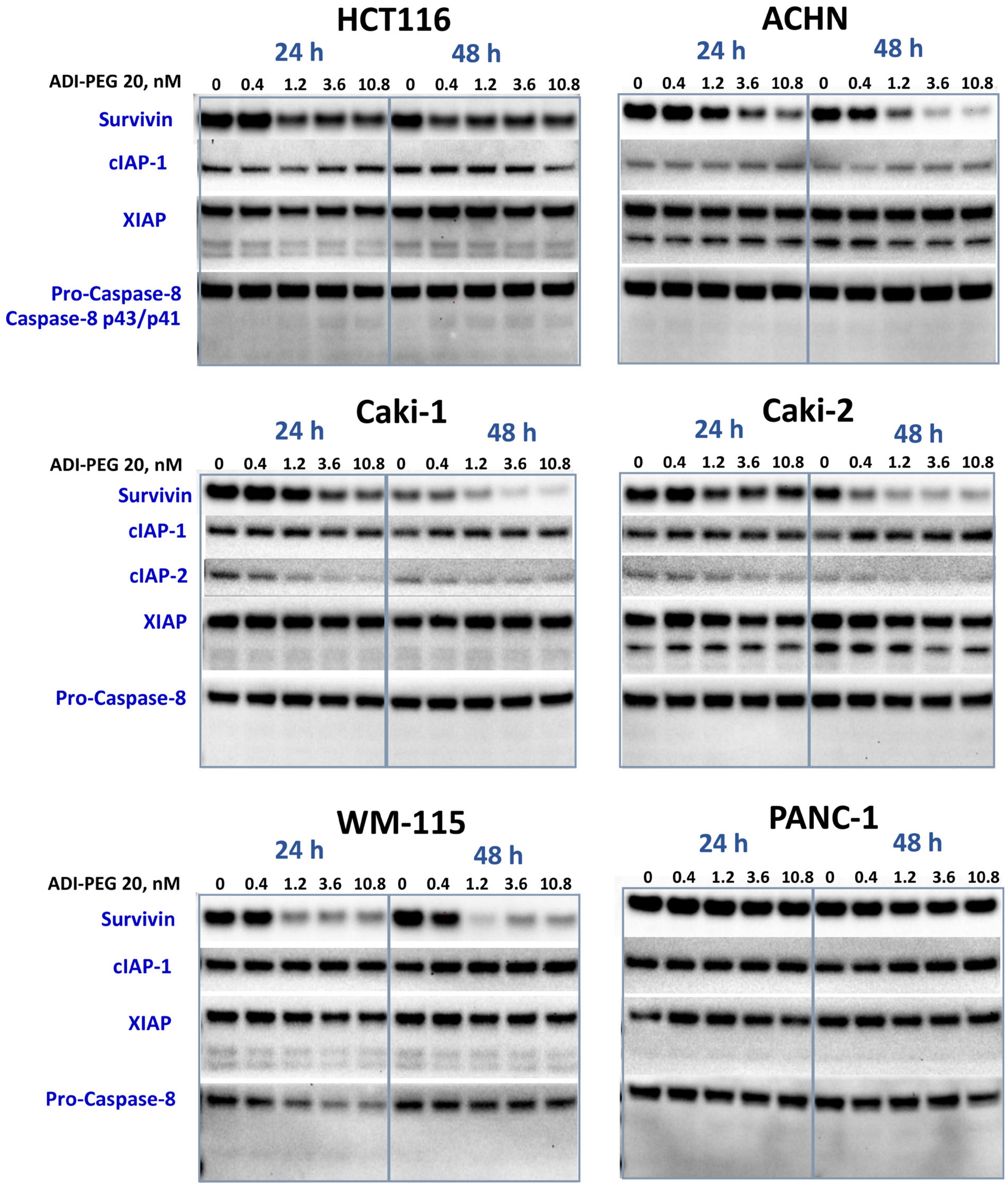

Supplementary Figure 2: Effect of 24 h and 48 h treatment with ADI-PEG 20 on survivin, cIAPs, XIAP and (pro)-caspase-8 in HCT116, ACHN, Caki-1, Caki-2, WM-115 and Panc-1 cell lines.

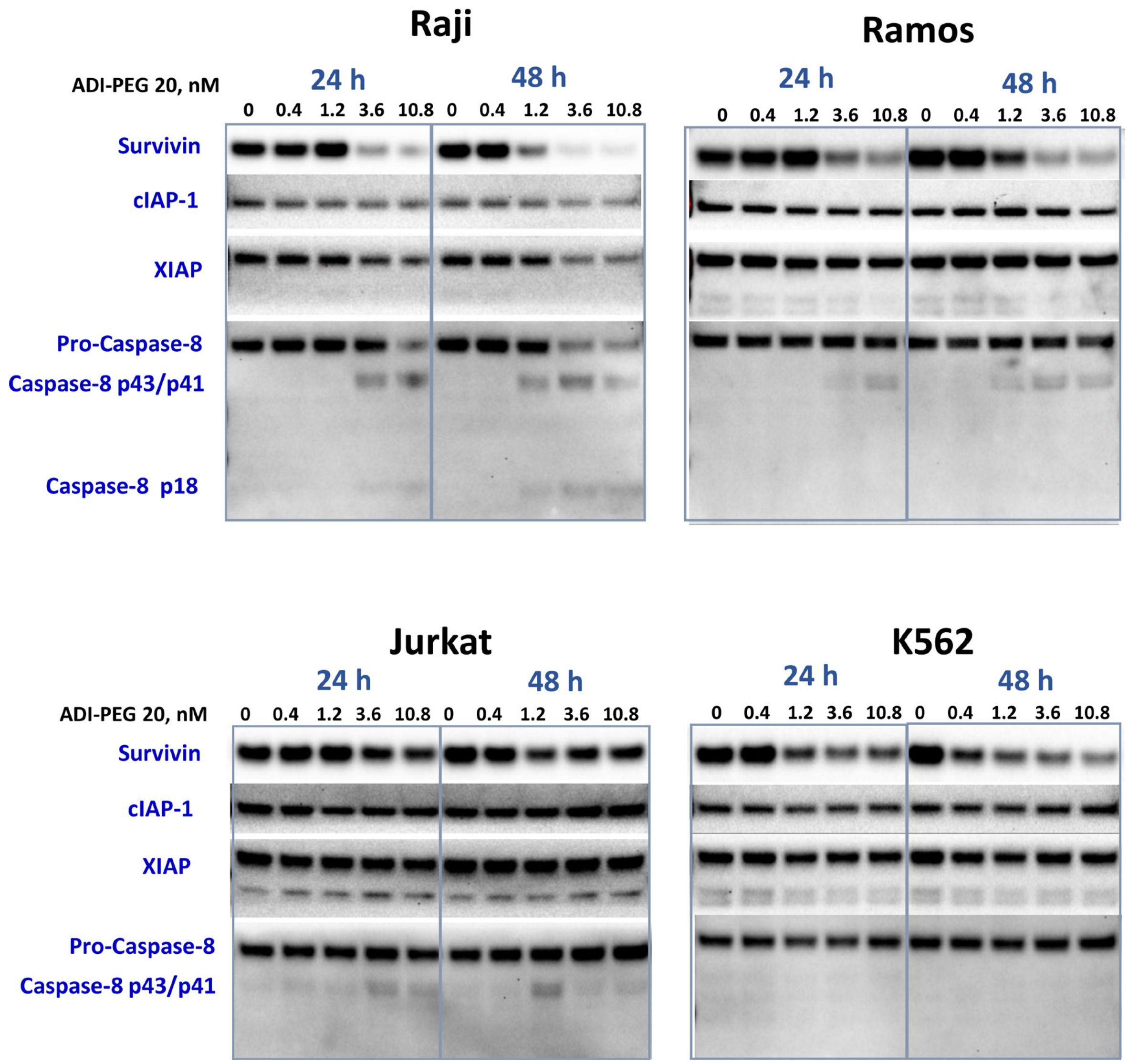

Supplementary Figure 3: Effect of 24 h and 48 h treatment with ADI-PEG 20 on survivin, cIAP-1, XIAP and (pro)-caspase-8 in Raji, Ramos, Jurkat and K562 cell lines.

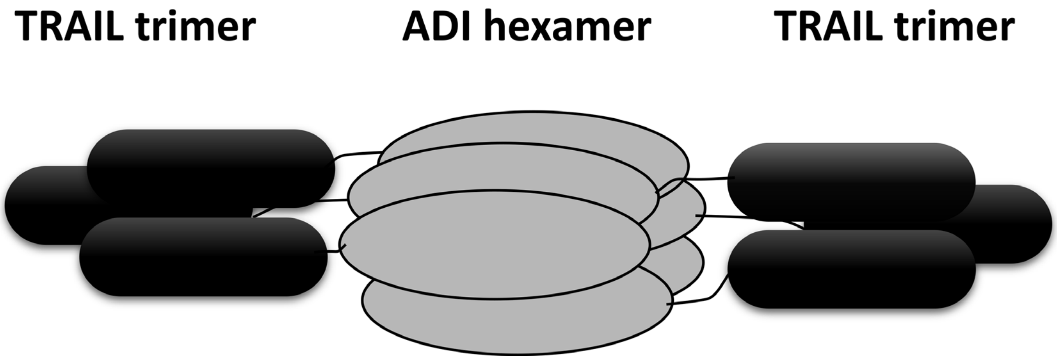

Supplementary Figure 4: Schematic representation of ADI-TRAIL fusion protein.

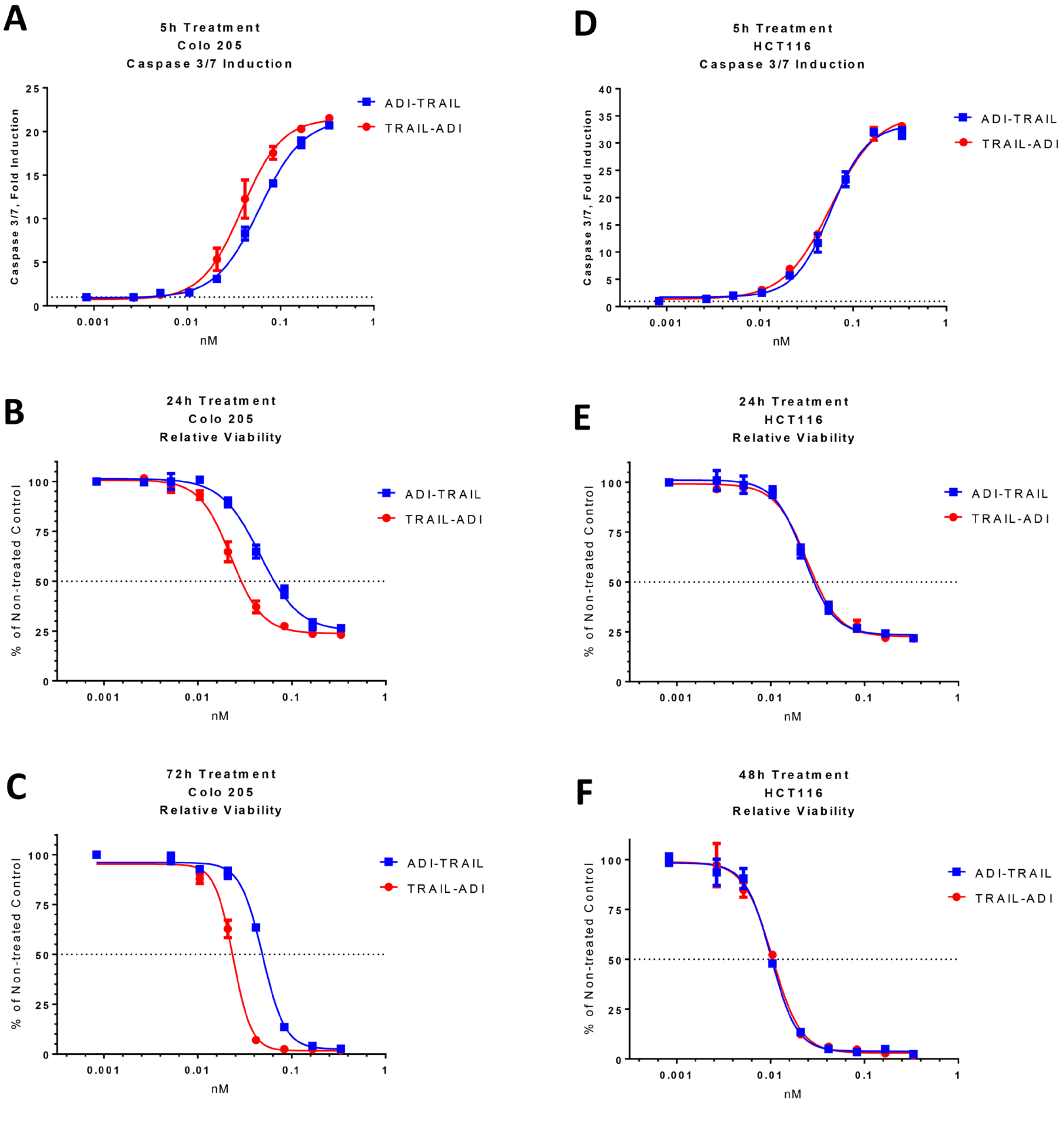

Supplementary Figure 5: Activity of ADI-TRAIL fusion protein versus TRAIL-ADI fusion protein. The effect of ADI-TRAIL (C-terminus of ADI is fused to N-terminus of TRAIL) was compared to that of TRAIL-ADI (N-terminus of ADI is fused to C-terminus of TRAIL) in ADI-non-sensitive cell line Colo 205 (**A**–**C**) and ADI-sensitive cell line HCT116 (**D**–**F**). Caspase 3/7 induction (A and D) was measured after 5h treatment and relative cell viability was assessed after 24 h (B and E) and 48 h (F) and 72 h (C).
